# The Effect of the Chitosan on Bleeding Control and Healing of Dog Buccal Mucosal Wound: An in Vivo Experimental Study

**DOI:** 10.22038/ijorl.2025.80060.3690

**Published:** 2025

**Authors:** Reza Kaboodkhani, Zohre Zandifar, Seyed Hossein Owji, Omid Koohi-Hosseinabadi, Seyed Mohammad Owji, Sarah Yousefi, Abbas Mohammadi Oshnari, Salman Taheri, Ali Asghar Mohammadi, Naeimeh Sadat Asmarian

**Affiliations:** 1 Otolaryngology Research Center, Department of Otolaryngology, Shiraz University of Medical Sciences, Shiraz, Iran.; 2 Otorhinolaryngologist and Head and Neck Surgeon, Private Practice, Shiraz, Iran.; 3 Otorhinolaryngology Research Center, Otorhinolaryngology Head and Neck Surgery Department, Imam Khomeni Hospital Complex, Tehran University of Medical Sciences, Tehran, Iran.; 4 Laparoscopy Research Center, Shiraz University of medical Science, Shiraz, Iran.; 5 Department of Pathology, School of Medicine, Shiraz University of Medical Sciences, Shiraz, Iran .; 6 Chemistry and Chemical Engineering Research Center of Iran (CCERCI), P.O. Box 14335-186, Tehran, Iran.; 7 Anesthesiology and Critical care Research Center, Shiraz University of Medical Sciences, Shiraz, Iran.

**Keywords:** Chitosan, Bleeding time, Mouth mucosa, Wound healing, Hemorrhage

## Abstract

**Introduction:**

Chitosan, which is an amino polysaccharide resulting from the deacetylation of chitin, regarding its biomedical features such as antioxidant activity, muco-adhesive and hemostatic properties, antibacterial and anti-inflammatory effects, used for medical purposes. Oral cavity and oropharynx are two important structures that in terms of rich blood supply, hemorrhage might be life threating requiring effective hemostatic management. In present study, we evaluated the effect of chitosan dressing on oral cavity wound healing and hemostasis.

**Materials and Methods:**

Nine male dogs were selected simple randomized and divided into three groups. A wound was made in the buccal mucosa bilaterally. We used chitosan powder dressing on the right side, chitosan-free gaze was used on the left side and bleeding time was determined. Three Dogs after5, three dogs after 10, and three dogs after15 days underwent biopsy bilaterally and pathologic assessment performed. Continuous and ordinal variables were reported as a median and IQR, and Wilcoxon test, and Friedman test were used to analyzing. Data were analyzed using SPSS 21.

**Results:**

Statistical analysis (Wilcoxon test) showed that the overall differences between two groups were statistically significant for (Acute inflammation score: P=.025; Collagenization score: P=.046; Neovascularization score: P=.046; Granulation tissue: P=0.046, and Re-epithelialization score: P=0.038). Chitosan powder dressing application significantly reduced acute inflammation and neovascularization, and increased collagenization, granulation tissue and re-epithelialization. Furthermore, the median time of bleeding and percentage change of wound size which were not statistically significant for all 3days in the case and control groups but they were clinically significant.

**Conclusion:**

Chitosan salt powder dressing positively impacts the wound bleeding control and on mucosal wound healing according to histopathologic and gross wound healing indexes.

## Introduction

Various synthetic materials were applied for wound healing and bleeding control so far. Although these components were effective for these purposes, they did not simultaneously have antimicrobial properties, high absorption, biological compatibility, and a moisturizing wound environment to facilitate wound healing and bleeding control (1). Among all natural materials used for this purpose, the chitosan is unique. Chitosan is an amino polysaccharide resulting from the deacetylation of chitin in crustacean waste, such as shrimp, crayfish, arthropods, and insect coticules (2,3). Regarding its exclusive biomedical features such as antioxidant activity, cholesterol and triglyceride trapping, muco-adhesive and hemostatic properties, and antibacterial and anti-inflammatory effects, widely used for medical purposes (4). Chitosan is used as a bandage and hydrogel in the biomedical field to help wound healing and hemorrhage control. Although its mechanism is not clear, according to the research, three kinds of actions of chitosan lead to hemostasis and wound healing, and it seems to be able to accelerate all stages of wound healing: plasma absorption coagulation of erythrocyte and adhesion of platelet (1,2,3,5). As a matter of fact, the hemostatic effect of chitosan is a result of plasma absorption following erythrocyte agglutination. Moreover, it promotes the function of inflammatory cells, including macrophages and leukocytes subsequent fibroblasts (6). Oral cavity and oropharynx are two important structures supplied by different branches of external carotid artery that in terms of rich blood supply, hemorrhage caused whether by injury or surgery might be life threating requiring effective hemostatic management (7,8). In present study, we evaluated the effect of chitosan dressing on oral cavity wound healing and hemostasis. 

## Materials and Methods

### Experimental Design and Surgical Procedure

This study is an in vivo experimental study from march 2021 to October 2021 at animal center affiliated to shiraz university of medical sciences, nine male dogs were selected based on previous similar animal study (9). Dogs were obtained from the animal center and were aged between two and three years. They were divided into three groups via simple random sampling; because they were nearly the same breed, age, and weight and were kept in the same conditions. After general anesthesia with ketamine 10% (3 - 4 cc) and xylazine (1- 1.5 cc) a wound was made in the buccal mucosa with surgical scissors bilaterally in the form of a circle with a diameter of 1 cm and a thickness of 2 mm. The right side is considered as case, and the left side is considered as control. We used chitosan powder dressing on the case side, and chitosan-free gaze was used on the control side. We determined the bleeding time of the case side until complete stop in seconds and compared it with the control side. Three Dogs after 5, three dogs after 10, and three dogs after 15 days underwent biopsy bilaterally, and after wound healing, they were released. Pathologic assessment performed and the result abstained are as follows. The humane treatment of research animals was considered.This study was approved by the institutional review board (IRB) of Shiraz University of Medical Sciences and the ethics committee (IR .SUMS. MED. REC.1400.114). 

### Chitosan preparation:

Extraction of chitosan from shrimp and other marine sources requires four steps:

1-demineralization: First of all, for whole calcium carbonate extracted from shrimp exoskeleton, 100-gram dried shrimp shell was diluted in dilute hydrochloric acid at room temperature for five hours, then it was filtered and washed with distilled water until the solution reached natural PH. It was dried at 60֯ C for 4 hours in an oven. In this case, the maximum amount of weight loss was observed at only 53 grams out of 100 grams remain. 

2-deproteinization: Deproteinization of shrimp shells requires placing the dry shells in a dilute sodium hydroxide (caustic soda).53 grams of demineralized powder remained from step one, was mixed in dilute caustic soda at 60֯ C for 2 hours to complete deproteinization. 

It was washed with distilled water until neutral PH reached, and dried at 60֯ C in an oven. The dried material obtained was chitin being 25 grams.

3-decolorization: As an oxidant, sodium hypochlorite may decolorize chitin. In this example, 25 grams of chitin are combined for 30 minutes with 5% hypochlorite sodium, then rinsed with ethanol and distilled water and dried in an oven.

4-Deacetylation: The conversion of chitin to chitosan was done by deacetylation. For this purpose, the chitin was placed in concentrated sodium hydroxide 50% at 90֯ C for 2 hours. 

Then, it was washed with distilled water and dried, and 12 grams chitosan was obtained. For chitosan film,1 gram of dried chitosan, mixed in acetic acid 1% for 2 hours until the chitosan dissolved in the acid. Then, this solution was left 24 hours to remove the bubbles. Poured some of the resulting viscous liquid into the petri dish and dried in the oven at 50֯ C for 3 hours. The film was separated from the dish surface by adding dilute sodium hydroxide and ethanol. The resultant film was sterilized in an incubator for 12 hours after being cleaned with distilled water. 

### Preparation of chitosan salts:

 To determine the wound healing ability and hemostatic properties of chitosan and its derivatives, three kind of chitosan salts prepared. The prominent properties of these salts are that compared to chitosan, they are water soluble gel in high concentration and absorb easily by tissues as well.

1-Preparation of acetate, ascorbate and lactate salts:

To synthesize of these salts, 5 g of purified chitosan with 92% de-acetylation was added to 100 cc of isopropanol 20 cc of 4M solution of each acids (lactic acid, acetic acid, ascorbic acid) was used then added to this complex and sterilized for 24 hours at room temperature. After that, the salts were purified and washed with isopropanol to remove the excess ions and dried at 50 ° C in an oven, the complex was placed in a dialysis bag for 2 days in distilled water.

2- Preparation of homeostatic gels:

 In order to investigate the homeostatic effect,3 gels with concentrations of 1%, 2% and 3% of each salts were prepared. None of them could stop bleeding.

3- Preparing the film using the prepared salts:

10 cc of gel with a concentration of 1% was prepared from each of the salts. 10 cc of 10% polyvinyl alcohol solution was prepared separately. 

The films were prepared in different ratios: chitosan salts and PVA in 30/70 and 50/50 ratios, and chitosan salts , PVA and glycerin in 10:30:60 ratios. These gels were placed in a petri dish and in an oven at a temperature of 50 degrees to dry the gels and turn them into a film. These films are absorbable. Finally, based on previous studies, chitosan absorbable salts in powder form have the best results in coagulation and wound healing. Among these powders, chitosan lactate salt showed better results than other salts; so, finally we used this salt.

### Histological Analysis:

Histologic features were studied in paraffin-embedded sections using hematoxylin and eosin and Masson’s trichrome stains at a magnification of ×40 to ×400. The main histologic findings included:

- Amount of acute and chronic inflammatory infiltration
- The amount and maturation of neovascularization, collagenization, granulation tissue, and re-epithelialization.

We used a modified scoring system (Table 1) for histologic evaluation based on the scoring system suggested by Abramov et al. (10).

**Table 1:** Grading of histologic indexes for wound healing

**Grade3**	**Grade2**	**Grade1**	**Grade 0**	**definition**	
Abundant	Moderate	Scant	None	Defined as the presence of neutrophils	Acute inflammation
Abundant	Moderate	Scant	None	Defined as the presence of plasma and monocytic cells	Chronic inflammation
thick, gross densely arranged collagen fibers seen throughout the wound area	thin, delicate loosely arranged collagen fibers are seen in the surface and center of the wound area, but thicker and gross in the deep and margins	thin, delicate loosely arranged collagen fibers seen throughout the wound area	None	------	Collagen deposition
More than 10 vessels per HPF	6–10 vessels per HPF	Up to five vessels per HPF	None	------	percentage of new vascularization
Fully matured	Moderate maturation	Mild maturation	Immature	Mature fibroblasts are thin and usually arranged in compacted parallel layers. Immature fibroblasts are stellate-shaped and less organized.	Granulation tissue
Complete and mature	Complete but immature or thin	Partial	None	------	Re-pithelialization

In this scoring, there is more than one determinant for some histologic parameters so that both amount and maturation of granulation tissue as well as acute and chronic inflammation were determined. cute inflammation was defined as the presence of the neutrophil, while chronic inflammation was defined as mononuclear cells, including lymphocytes and plasma cells. The shape and orientation of fibroblasts determined the degree of granulation tissue maturation. Mature fibroblasts are thin and usually arranged in compacted parallel layers, and immature fibroblasts are stellate-shape and not well organized. We used Masson’s trichrome stains to increase the accuracy of detection of immature and mature collagen bundles and so better collagen deposition scoring. The above histological features were evaluated in sections of buccal mucosa wounding in 5 days, 10 days, and 15 days and also in the right side (chitosan) and the left side (control) and comparing the above parameters.

### Wound size analysis:

A high-quality camera was used to photograph 

all primary and secondary wound sites. The photographs were then processed using image j software (National Institutes of Health, Bethesda, MD, USA) to determine the precise area of the wound in square centimeters. In photographs, a sterile ruler adjacent to the wound site was used as a measurement scale.

### Statistical Analyses:

Continuous and ordinal variables were reported as a median and IQR, and Wilcoxon test, and Friedman test were used to analyzing. Plots were displayed by Graph pad prism (version 6). Data were analyzed using SPSS 21, and p-values < 0.05 were considered statistically significant.

## Results

The effects of chitosan powder dressing on mucosal wound healing based on histologic criteria of acute inflammation, chronic inflammation, collagenization, neovascularization, granulation tissue, and re-epithelialization are summarized in Table 2.

**Table 2:** Number of Cases in different acute inflammation, Chronic inflammation, collagenization, neovascularization, granulation tissue and re-epithelialization grades for each group.

			**Grade**			
**Variable**	**day**	**group**	**0**	**1**	**2**	**3**
Acute inflammation	5	case	0	0	2	1
		Control	0	0	0	3
	10	case	0	2	1	0
		Control	0	0	2	1
	15	case	3	0	0	0
		Control	3	0	0	0
Chronic inflammation	5	case	3	0	0	0
		Control	3	0	0	0
	10	case	0	1	2	0
		Control	0	0	1	2
	15	case	0	3	0	0
		Control	0	1	2	0
collagenization	5	case	2	1	0	0
		Control	2	1	0	0
	10	case	0	1	2	0
		Control	0	2	1	0
	15	case	0	0	2	1
		Control	0	2	1	0
Neovascularization	5	case	0	0	0	3
		Control	0	0	0	3
	10	case	0	1	2	0
		Control	0	0	2	1
	15	case	0	3	0	0
		Control	0	1	2	0
Granulation tissue	5	case	3	0	0	0
		Control	3	0	0	0
	10	case	0	1	2	0
		Control	0	3	0	0
	15	case	0	0	1	2
		Control	0	0	3	0
Re-epithelialization	5	case	3	0	0	0
		Control	3	0	0	0
	10	case	0	1	2	0
		Control	1	2	0	0
	15	case	0	0	0	3
		Control	0	2	1	0

It represents the grade frequency distribution of acute inflammation, chronic inflammation, collagenization, neovascularization, and granulation tissue, and re-epithelialization indexes across case and control groups on different 3 groups (5, 10 and 15 days). For example, number 3 in the acute inflammation variable for the case group on day 15th, indicates that all three samples were detected with a grade of 0. Statistical analysis (Wilcoxon test) showed that the overall differences between two groups were statistically significant for (Acute inflammation score : P = .025; Collagenization score: P= .046; Neovascularization score: P= .046; Granulation tissue: P=0.046, and Re-epithelialization score: P=0.038).Table 3 shows median grade scores of the acute inflammation, Chronic inflammation, collagenization, neovascular- rization, granulation tissue and re-epithelialization indexes which are merged for all 3 days in the case and control groups. 

Chitosan powder dressing application significantly reduced acute inflammation and neovascularization, and increased Collagenization, granulation tissue and re-epithelialization. Furthermore, table 3 shows the median of time of bleeding and percentage change of wound size which were not statistically significant for all 3 days in the case and control groups; but they were clinically important. This indicates that the difference in mean values between the case and control groups was clinically significant (e.g., the mean bleeding time was 138 seconds in the case group and 309 seconds in the control group), but due to the small sample size, this difference did not reach statistical significance.

**Table 3:** The comparison of time of bleeding, percentage change of wound size, acute inflammation, chronic inflammation, collagenization, neovascularization, granulation tissue and re-epithelialization between case and control groups.

**Variables**	**Case Median (IQR)**	**Control Median (IQR)**	**P-value**
Time of bleeding, second	138(74-345)	309(141-458)	0.139
percentage of reduction of wound size	69.63±22.16	52.66±32.10	0.210
Acute inflammation	1(0-2)	2(0-3)	0.025
Chronic inflammation	1(0-1.5)	2(0-2.5)	0.059
Collagenization	2(0.5-2)	1(0.5-1.5)	0.046
Neovascularization	2(1-3)	2(2-3)	0.046
Granulation tissue	2(0-2.5)	1(0-2)	0.046
Re-epithelialization	2(0-3)	1(0-1)	0.038

**Fig 1:**
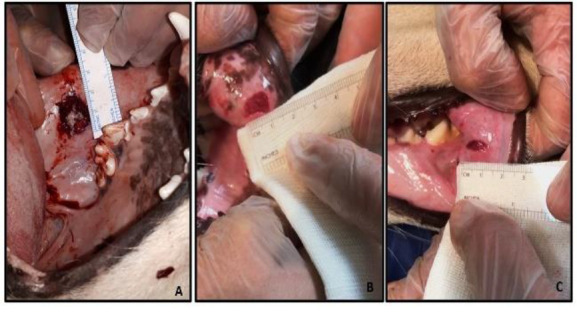
primary wound covered with chitosan powder dressing (A), wound after 10 days in both control (B) and case (C) groups. The wound size is smaller in case group.

Fig. 1 shows primary wound and also wound after 10 days in both case and control groups which the wound size is smaller in case group.

**Fig 2:**
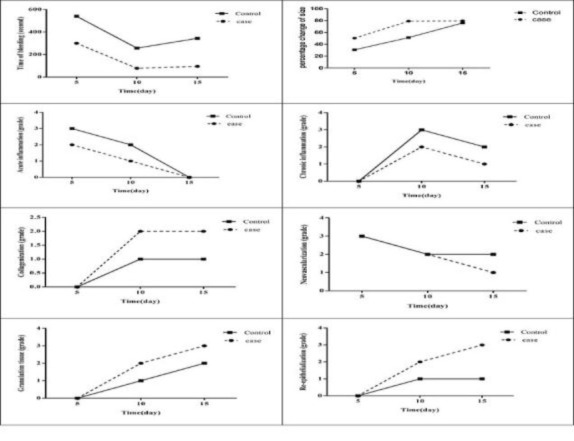
The comparison of median of time of bleeding, percentage change of wound size, acute inflammation, chronic inflammation, collagenization, neovascularization, granulation tissue and re-epithelialization during fifth, tenth, and fifteenth days.

The figures indicate a decrease in bleeding time, secondary wound size, acute inflammation, and neovascularization over time, as well as an increase in granulation tissue and re-epithelialization, between the case and control groups following chitosan powder dressing application, but these differences were not statistically significant (Fig. 2). Moreover, based on the Friedman test, the time effect was not significant in all of the variables in the case and control groups.

### Histopathologic results:

**Fig 3:**
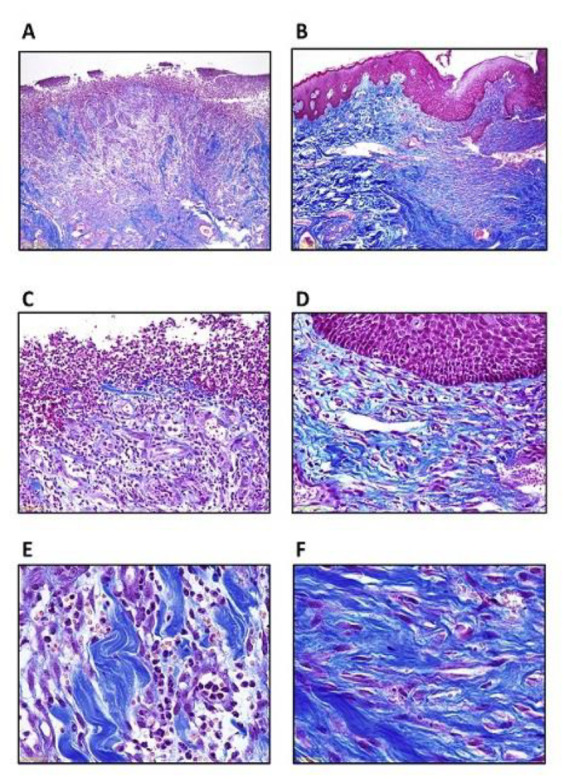
Photomicrographs of histologic sections of dog buccal mucosa 5 days after wounding in the control and chitosan groups showing maximum degree of acute inflammatory cells mostly on the surface of the wound, as the compact dense bond-like pattern. The amount of immature granulation tissue increased in both groups, but in the chitosan group, few thin delicate immature collagen fibers formation is seen in the dermis. No re-epithelialization is seen in both groups. (Masson’s trichrome. control group: A, C, E and chitosan group: B, D, F)

**Fig 4:**
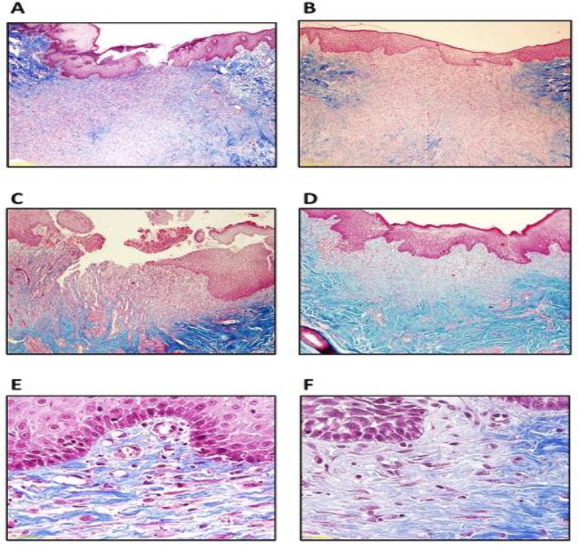
Photomicrographs of dog buccal mucosa histologic sections 10 days after wounding in the control and chitosan groups. Note beginning of re-epithelialization of stratified squamous epithelium in chitosan group (B and D). But, in the control group, still dense compact acute inflammatory cells are present on the surface of the wound (A and C). Dermis show loosely arranged thin delicate collagen fibers in the control group; while in the chitosan group, collagen fibers are thicker and more mature. (Masson’s trichrome. control group: A, C, E and chitosan group: B, D, F)

**Fig 5:**
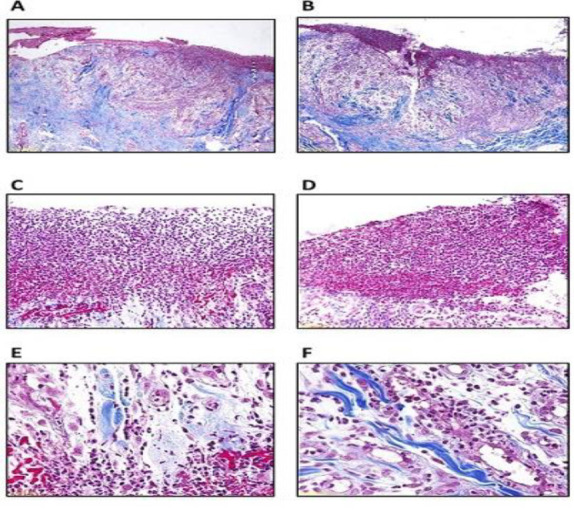
Photomicrographs of dog buccal mucosa histologic sections 15 days after wounding in the control and chitosan groups. The original wound is covered completely with a thin stratified squamous epithelium in the chitosan group, but re-epithelialization is defective in the control group. The collagen fibers are thick and appear densely arranged and more compact via the dermis of the wound area in the chitosan group compared to the control group, which shows thin, delicate, loosely arranged collagen fibers through the wound dermis. (Masson’s trichrome. control group: A, C, E and chitosan group: B, D, F)

Acute inflammation increased after wounding on day 5 in the dogs’ buccal mucosa and declined in the chitosan group at day 10; while still is present in control group at day 10. Chronic inflammation was not present at day 5 in both groups, increased at 10^th^ day and decreased, but still present on day 15 in the control group; while in chitosan is scanty. The amount of immature granulation tissue increased significantly after wounding of buccal mucosa on day 5 in chitosan and control groups and declined on day 10 and day 15. In contrast, the maturation of granulation tissue was slightly increased on day 10 and even more increased on day 15 in the chitosan group than control. Neovascularization scores significantly increased after wounding on 5^th^ day and decreased on 10^th^ day which were scant on day 15 in the control group. This decline in neovascularization was more remarkable in the chitosan group on day 10 and 15 (Fig. 3,4,5).

On 5^th^ day, re-epithelialization of stratified squamous epithelium is not seen in the control and chitosan groups.

On 10^th^ days, re-epithelialization is delayed in the control group compared to the chitosan group, which shows the beginning of re-epithelialization.

On 15^th^ days, in the chitosan group, the original wound was completely covered with stratified squamous epithelium, but the thickness of epithelium is not as much as the adjacent normal epithelium. The re-epithelialization in the control group is defective compared to chitosan group (Fig. 5).

## Discussion

Chitosan, a natural polymer derived from shelled crustaceans, has significant biological properties, including biodegradability, biocompatibility, antimicrobial activity, and non-toxicity. This material is manufactured in a variety of ways, including scaffolds, films, gaze, powder, and hydrogel.

We assessed the effect of chitosan powder dressing application on wound healing and bleeding control of wounds. Results indicated that histopathologic wound healing indexes, such as acute inflammation, neovascularization, collagenization, granulation tissue, and re-epithelialization were improved overall after merging 3 gropes which was statistically significant. But, by considering separate time groups (5-, 10-, and 15-days follow-up), although these differences were not statistically significant, they were clinically significant. It could be in terms of the small sample size which can affect statistical analysis. Moreover, similar to the above, the percent of wound size reduction and wound site bleeding time were reduced clinically, although these differences were not statistically significant in terms of sample size limitations.

Our results show that in case groups, bleeding times after creating the wound were clinically shorter than in control groups (median: 138 seconds versus 309 seconds). It means that the chitosan powder dressing could control bleeding better than the usual dressing. Control of bleeding and as a result reducing bleeding time is another important property of this material. Previous studies showed the role of different forms of chitosan in bleeding control in different bleeding sites. For example, in the field of gynecology, Carles et al. show that chitosan gauze and powder reduced different forms of serious post-partum bleeding (11). Another similar study revealed the positive effect of chitosan gaze packing on post-hysterectomy persistent bleeding and chitosan powder in vaginal bleeding in terms of multiple tears (12). Ishizuka et al. evaluated the ability of submucosal injection of chitosan to reduce bleeding after mucosal resection of rat stomach (13). They found that the total amount of bleeding 5, 10, 15, and 20 min after mechanical mucosal resection was significantly lower in the chitosan injection group compared to the hypertonic saline injection group. Past studies also assessed the application of chitosan in the control of Sinonasal cavity bleeding. Chung et al. through a randomized double-blind controlled trial evaluated postoperative bleeding after endoscopic sinus surgery (14). Their results showed that chitosan gel led to rapidly complete hemostasis compared to the control side. Consistent with such findings, Zhou et al. done a systematic review and meta-analysis to evaluate the efficacy of chitosan dressing in endoscopic sinus surgery and found that chitosan dressing could significantly improve the hemostasis (15). On the other hand, its application is not unlimited to all sites of hemorrhage. In a recent study, Deineka et al. indicated that chitosan is not recommended for visceral application (16). Because it can lead to moderate inflammation which has slow degradation. However, in our study, there were no signs of abnormal inflammation, reaction or anaphylaxis. Overall, in our study, we used chitosan power in buccal mucosa wound. In a previous study, less attention was paid to its application to mucosal bleeding. 

We found that wound size was more reduced in the chitosan group compared to the control group. We considered the percent of wound size reduction as an approximate gross index for the assessment of wound healing. In line with our result, in a study by Ishizuka et al., the amount of wound size reduction on the seventh day after mucosal resection was more in the chitosan group (13). In another study by Zhao et al., the rat model compared skin wound healing in the chitosan calcium alginate dressing with just calcium alginate dressing (17).They found that in the chitosan group, wound size and area were significantly smaller than in the calcium alginate group on the 3rd, 7th, and 14th post-operative days. Furthermore, the time of complete healing and wound closer was significantly shorter in the chitosan group. Chen et al., in another similar research, assessed the effect of chitosan on skin wound healing in the animal models (18). They figured out that chitosan groups had significantly greater wound contraction on days 3, 5, and 7 after the operation than the negative control group. Overall, in line with our results, previous research showed that chitosan dressing could promote wound healing and decrease wound area faster.

Another important finding of our study is that wound healing was improved in the chitosan group in comparison to the control group. In support of our findings, some research showed that chitosan can accelerate the wound repair process. For instance, a study determined the impact of chitosan submucosal injection on post mucosal resection wound healing (13). The results revealed that wound healing was raised by the usage of chitosan, although the effect was not statistically significant. Besides, wound healing was slightly faster in the chitosan groups than in the hypertonic saline group during seven-day follow-up. In a recent review article by Saini et al., immunomodulatory and wound healing properties of chitosan were appraised (19). They found that many reviewed studies suggested that chitosan stimulates the release of anti-inflammatory cytokines, chemokines, and growth factors in terms of its considerable immuno-stimulatory activities. Therefore, it has a helpful influence on all phases of wound healing, including hemostasis, inflammation, proliferation, cell migration, and cell regeneration. Another research revealed that chitosan curcumin mouthwash was effective in the management of oral inflammatory ulcers (20). Tavaria et al. through in vivo study in rats showed that chitosan was not cytotoxic and led to good skin wound healing, even in diabetic animals (21). Results of another research showed that chitosan hydrogels by increasing wound angiogenesis and inhibiting inflammation could improve skin wound healing (18). In addition to skin and mucosal wound healing, some previous studies indicated that chitosan has a positive effect on healing of other tissue such as bone. For example, Filho et al. found that chitosan-gelatin osteoconductive matrix increases bone repair after causing an intrabuccal bone defect in rat (22). The same as the previous article, the results of another study by Gupta et al. revealed that chitosan increased wound healing and early osteogenesis in the erupted tooth sockets after extraction (23). The strength of this study is that the effect of chitosan on the buccal mucosal wounds was evaluated, while most previous studies have focused on the effect of this material on skin wounds. Moreover, a big animal such as a dog was used to better assess the healing status of mucosal wounds. While in many past studies, smaller animals such as rats were used.

A number of limitations need to be considered. For instance, as mentioned above, some of our results, although were clinically significant, were not statistically significant in terms of the low sample size. Hence, more research with larger sample size is necessary to validate the conclusions drawn from this study. Furthermore, we assessed the effect of powder type of chitosan as a dressing for the mucosal wounds. So, future research should concentrate on another type of chitosan dressing, such as hydrogel, solution, and gaze form. 

## Conclusion

 Chitosan powder dressing application positively impacts the wound bleeding control and on mucosal wound healing according to histopathologic and gross wound healing indexes.

## References

[1] Mehrabani MG, Karimian R, Rakhshaei R, Pakdel F, Eslami H, Fakhrzadeh V (2018). Chitin/silk fibroin/ TiO(2) bio-nanocomposite as a biocompatible wound dressing bandage with strong antimicrobial activity. International journal of biological macromolecules.

[2] Nwe N, Furuike T, Tamura H (2014). Isolation and characterization of chitin and chitosan from marine origin. Advances in food and nutrition research.

[3] McGrath B, McCarthy S, Sam K, Wold A, Stolten M, Bennett A (2017). Biocompatible and bioabsorbable derivatized chitosan compositions. Google Patents.

[4] Ghimire S, Sarkar P, Rigby K, Maan A, Mukherjee S, Crawford KE (2021). Polymeric Materials for Hemostatic Wound Healing. Pharmaceutics..

[5] Feng P, Luo Y, Ke C, Qiu H, Wang W, Zhu Y (2021). Chitosan-Based Functional Materials for Skin Wound Repair: Mechanisms and Applications. Frontiers in bioengineering and biotechnology.

[6] Mizuno K, Yamamura K, Yano K, Osada T, Saeki S, Takimoto N (2003). Effect of chitosan film containing basic fibroblast growth factor on wound healing in genetically diabetic mice. Journal of Biomedical Materials Research Part A: An Official Journal of The Society for Biomaterials, The Japanese Society for Biomaterials, and The Australian Society for Biomaterials and the Korean Society for Biomaterials.

[7] Mun MJ, Lee C-H, Lee B-J, Lee J-C, Jang JY, Jung SH (2016). Histopathologic Evaluations of the Lingual Artery in Healthy Tongue of Adult Cadaver. Clin Exp Otorhinolaryngol.

[8] Shahbazi A, Grimm A, Feigl G, Gerber G, Székely AD, Molnár B (2019). Analysis of blood supply in the hard palate and maxillary tuberosity-clinical implications for flap design and soft tissue graft harvesting (a human cadaver study). Clinical oral investigations.

[9] Iravani K, Mehravar S, Bahador M, Azarpira N (2021). The Healing Effect of Amniotic Membrane in Laryngeal Defects in Rabbit Model. The Laryngoscope.

[10] Abramov Y, Golden B, Sullivan M, Botros SM, Miller JJ, Alshahrour A (2007). Histologic characterization of vaginal vs abdominal surgical wound healing in a rabbit model Wound repair and regeneration : official publication of the Wound Healing Society. European Tissue Repair Society.

[11] Carles G, Dabiri C, McHirgui A, Saoudi EO, Hcini N, Pouget K (2017). Uses of chitosan for treating different forms of serious obstetrics hemorrhages. Journal of gynecology obstetrics and human reproduction.

[12] Carles G, Dabiri C, Mchirgui A (2016). Different use of Chitosan for treating serious obstetric hemorrhages. Gynecol Obstet Res Open J.

[13] Ishizuka T, Hayashi T, Ishihara M, Yoshizumi Y, Aiko S, Nakamura S (2007). Submucosal injection, for endoscopic mucosal resection, of photocrosslinkable chitosan hydrogel in DMEM/ F12 medium. Endoscopy.

[14] Chung Y-J, An S-Y, Yeon J-Y, Shim WS, Mo J-H (2016). Effect of a Chitosan Gel on Hemostasis and Prevention of Adhesion After Endoscopic Sinus Surgery. Clin Exp Otorhinolaryngol.

[15] Zhou JC, Zhang JJ, Zhang W, Ke ZY, Zhang B (2017). Efficacy of chitosan dressing on endoscopic sinus surgery: a systematic review and meta-analysis. European archives of oto-rhino-laryngology : official journal of the European Federation of Oto-Rhino-Laryngological Societies (EUFOS) : affiliated with the German Society for Oto-Rhino-Laryngology - Head and Neck Surgery.

[16] Deineka V, Sulaieva O, Pernakov N, Radwan-Pragłowska J, Janus L, Korniienko V (2021). Hemostatic performance and biocompatibility of chitosan-based agents in experimental parenchymal bleeding. Materials science & engineering C, Materials for biological applications.

[17] Zhao WY, Fang QQ, Wang XF, Wang XW, Zhang T, Shi BH (2020). Chitosan-calcium alginate dressing promotes wound healing: A preliminary study. Wound repair and regeneration : official publication of the Wound Healing Society [and] the European Tissue Repair Society.

[18] Chen X, Zhang M, Wang X, Chen Y, Yan Y, Zhang L (2017). Peptide-modified chitosan hydrogels promote skin wound healing by enhancing wound angiogenesis and inhibiting inflammation. American journal of translational research.

[19] Saini S, Dhiman A, Nanda S (2020). Immunomodulatory Properties of Chitosan: Impact on Wound Healing and Tissue Repair. Endocrine, metabolic & immune disorders drug targets.

[20] Mahattanadul S, Mustafa MW, Kuadkaew S, Pattharachayakul S, Ungphaiboon S, Sawanyawisuth K (2018). Oral ulcer healing and anti-Candida efficacy of an alcohol-free chitosan-curcumin mouthwash. European review for medical and pharmacological sciences.

[21] Tavaria F, Jorge MP, Ruiz LT, Pintado M, Carvalho JE (2016). Anti-proliferative, anti-inflammatory, anti-ulcerogenic and wound healing properties of chitosan. Current Bioactive Compounds.

[22] Filho LBC, Silva GAB, Goes AM, de Abreu FAM, Assis MHS, Oliveira ASD (2021). Chitosan-based biomaterial and hyaluronic acid on the repair of intrabuccal bone defects in rats. Journal of the International Academy of Periodontology.

[23] Gupta A, Rattan V, Rai S (2019). Efficacy of Chitosan in promoting wound healing in extraction socket: A prospective study. Journal of oral biology and craniofacial research.

